# Population pharmacokinetics of remifentanil in critically ill patients receiving extracorporeal membrane oxygenation

**DOI:** 10.1038/s41598-017-16358-6

**Published:** 2017-11-24

**Authors:** Seungwon Yang, Hayeon Noh, Jongsung Hahn, Byung Hak Jin, Kyoung Lok Min, Soo Kyung Bae, Jiseon Kim, Min Soo Park, Taegon Hong, Jin Wi, Min Jung Chang

**Affiliations:** 1 Department of Pharmacy and Yonsei Institute of Pharmaceutical Sciences, College of Pharmacy, Yonsei University, Incheon, Republic of Korea; 2 Department of Pharmaceutical Medicine and Regulatory Sciences, Colleges of Medicine and Pharmacy, Yonsei University, Incheon, Republic of Korea; 3 Deparment of Clinical Pharmacology, Severance Hospital, Yonsei University College of Medicine, Seoul, Republic of Korea; 4 College of Pharmacy, The Catholic University of Korea, Bucheon, Kyunggido Republic of Korea; 5 Department of Pediatrics, Yonsei University College of Medicine, Seoul, Republic of Korea; 6 Division of Cardiology, Department of Internal Medicine, Yonsei University College of Medicine, Seoul, Republic of Korea

## Abstract

Extracorporeal membrane oxygenation (ECMO) is associated with pharmacokinetic (PK) changes of drugs. It presents considerable challenges to providing optimal dosing regimens for patients receiving ECMO. We aimed to describe the population PK of remifentanil in critically ill adult patients receiving venoartrial extracorporeal membrane oxygenation (VA-ECMO) and to identify determinants associated with altered remifentanil concentrations. The population PK model of remifentanil was developed using nonlinear mixed effects modelling (NONMEM). Fifteen adult patients who received a continuous infusion of remifentanil during VA-ECMO participated in the study. The PK of remifentanil was best described by a one-compartment model with additive and proportional residual errors. Remifentanil concentrations were affected by sex and ECMO pump speed. The final PK model included the effect of sex and ECMO pump speed on clearance is developed as followed: clearance (L/h) = 366 × 0.502^sex^ × (ECMO pump speed/2350)^2.04^ and volume (L) = 41. Remifentanil volume and clearance were increased in adult patients on VA-ECMO compared with previously reported patients not on ECMO. We suggest that clinicians should consider an increased remifentanil dosing to achieve the desired level of sedation and provide a dosing regimen according to sex and ECMO pump speed.

## Introduction

Extracorporeal membrane oxygenation (ECMO) has been increasingly used over the last decade to augment gas exchange and hemodynamic support in critically ill patients with refractory cardiac or respiratory failure^1,2^. With more patients being treated with ECMO, there is an increasing requirement to understand the complicated alterations in drug pharmacokinetics (PK) associated with the introduction of the ECMO system. Critically ill patients on ECMO receive multiple drugs, including sedatives, analgesics, antibiotics, and other drugs, to support their circulation and underlying medical conditions. The presence of ECMO increases the PK variability of these drugs owing to additional extracorporeal circulation^3^. With these dramatic PK alterations, which can lead to changes in drug concentrations, the provision of optimal pharmacotherapy to patients on ECMO remains a considerable challenge in clinical settings.

Because ECMO is often complicated by hemodynamic instability in critically ill patients with refractory cardiogenic shock, patients on VA-ECMO require adequate sedation and analgesia to minimize oxygen consumption, facilitate ventilation, alleviate patient stress responses and delirium, minimize the risk for catheter malposition, and prevent device dislodgement^4^. Remifentanil is one of the widely used sedative agents in ICU patients. Remifentanil is a lipophilic, selective μ-opioid receptor agonist that gives intense sedation and analgesia with a quick onset and short duration^5^. The elimination half-life varies from 10 to 20 min and is unaffected by hepatic or renal function^6^. Remifentanil rapidly equilibrates between blood and the site of drug effect, suggesting that the drug responses are correlated to the blood concentrations, which could be predicted by PK parameters^7^.

Anticipating the changes of PK in patients on ECMO is important to provide and guide a rational dosing regimen. Previous studies reported that PK alterations of drugs induced by the ECMO system are associated with an increased volume of distribution (V) and modified clearance (CL)^8,9^. PKs of drugs may be altered as a result of drug adsorption, sequestration, and inactivation in ECMO circuit components^10^. The varying degree of binding to the circuit components depends on the physiochemical properties of the drug, such as molecular size, lipophilicity, and plasma protein binding^11^. Current knowledge regarding PK alterations of drugs in patients on ECMO is limited, making it difficult to provide optimal dosing regimens of drugs to this patient population. The majority of PK data were limited to neonatal or pediatric patients. To date, although small PK studies of analgosedatives, including midazolam^12^, morphine^8^, propofol^13^, and fentanyl^14^, in ICU patients on ECMO have been emerging, there have been no PK studies regarding remifentanil during ECMO despite its frequent use.

Population PK analysis is a pragmatic approach to describe the drug PK, identify patient-related and clinical PK variability, and recommend appropriate dosing regimens based on model simulations.

Therefore, we investigated the population PK of remifentanil in critically ill adults receiving VA-ECMO and identified significant covariates associated with remifentanil exposure.

## Results

### Patient characteristics

Fifteen patients who received remifentanil by continuous infusion during ECMO support participated in the study. Table 1 displays select demographics, infusion rates, body mass index (BMI), total protein, indications for ECMO, duration of ECMO, and use of CRRT during ECMO for each patient. A higher proportion of study participants were male (67%). The median age, weight, BMI, and total protein were 57 years (interquartile range [IQR], 45, 69 years), 65.4 kg (IQR, 54.5, 70.0 kg), 23.8 kg/m^2^ (IQR, 21.2, 24.2 kg/m^2^), and 4.7 g/dL (IQR, 3.9, 5.3 g/dL), respectively. The patients received a median remifentanil infusion rate of 0.35 mg/h (IQR, 0.25, 0.35 mg/h). The median duration of ECMO support was 143 h (IQR, 96, 250 h) with median ECMO pump speeds of 2350 RPM (IQR, 2302, 2532 RPM). The most prevalent admission diagnosis to an ICU included acute myocardial infarction (n = 5) and non-ST-segment myocardial infarction (n = 4). Ten patients (67%) received concomitant CRRT during ECMO support.

**Table 1:** Individual baseline characteristics of study patients (n = 15).

Patient	Sex	Age (years)	Weight (kg)	BMI (kg/m^2^)	Total protein (g/dL)	Infusion rates (mg/h)	Indications for VA-ECMO	Duration of VA-ECMO (h)	Presence of CRRT
1	M	69	69.6	26.1	5.3	0.50 (0.50–0.70)	NSTEMI	250	Yes
2	M	62	53.8	18.6	5.3	0.40	Ischemic cardiomyopathy	137	No
3	F	55	60.6	22.2	3.3	0.14 (0.14–0.20)	Acute MI	250	Yes
4	F	76	40.8	18.1	3.0	0.30 (0.30–0.35)	Acute MI	57	Yes
5	M	63	69.0	23.9	3.9	0.25 (0.25–0.35)	Acute MI	139	Yes
6	M	52	70.0	24.2	4.6	0.35 (0.25–0.35)	Acute MI	76	Yes
7	M	19	69.9	24.2	3.4	0.35 (0.15–0.35)	Pulmonary embolism	96	No
8	M	78	53.0	22.9	4.7	0.35	NSTEMI	234	No
9	F	73	54.5	21.2	4.2	0.25 (0.25–0.35)	NSTEMI	48	No
10	M	57	94.0	31.8	5.0	0.35	Coronary artery occlusive disease	312	No
11	M	36	60.0	20.5	5.5	0.35 (0.15–0.35)	Myocarditis	106	No
12	F	47	65.4	24.3	6.2	0.35	STEMI	143	Yes
13	M	35	73.0	23.8	5.4	1.0	A. Fib, Bronchiolitis	532	Yes
14	F	57	57.0	22.0	4.4	0.35	Angina pectoris	168	Yes
15	M	45	73.2	24.2	5.0	0.20	Acute MI	257	Yes
Median		57	65.4	23.8	4.7	0.35		143	
IQR		45‒69	54.5‒70.0	21.2‒24.2	3.9‒5.3	0.25‒0.35		96‒250	

Data are expressed as count or median (range). A. Fib, atrial fibrillation; BMI, body mass index; CRRT, continuous renal replacement therapy; ECMO, extracorporeal membrane oxygenation; F, female; IQR, interquartile range; h, hours; MI, myocardial infarction; M, male; NTSEMI, non-ST-segment myocardial infarction; STEMI, ST-segment myocardial infarction.

### Population PK analysis

Fifty-five remifentanil concentrations (at least three samples from each of the 15 patients) were included in the analysis. The observed serum concentration-time profiles of remifentanil were best described by a one-compartment model with a zero-order input and first-order output (linear elimination). The interindividual variability was estimated only for CL as the addition of interindividual variability to V did not improve the fit. The estimate of the interindividual variability on V was near zero likely because of the narrow weight range in the patient population. Residual variability was best described by a combined (proportional and additive) error model. The eta shrinkage for CL in the base model was small (6%), confirming that our estimates were not over-parameterized.

**Figure 1:**
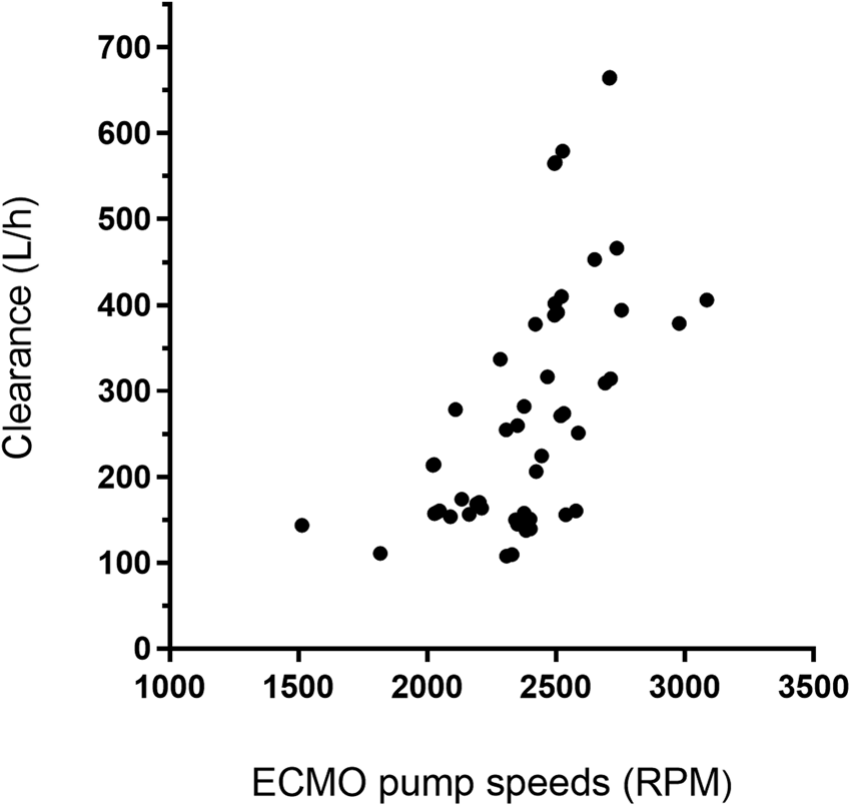
Plots of Bayesian posterior estimates of clearance (CL) with a covariate. The individual Bayesian estimates of CL as a function of extracorporeal membrane oxygenation (ECMO) pump speeds (dots).

Sex and the ECMO pump speed were significant covariates that statistically improved the base model when added to remifentanil CL (ΔOFV = −6.882 for sex; ΔOFV = −4.278 for the ECMO pump speed). Plots of Bayesian posterior estimates of parameters showed a linear relationship between remifentanil CL and the ECMO pump speed (Fig. 1). The final model including the effect of sex and ECMO pump speed on CL was as follows: CL (L/h) = 366 × 0.502^sex^ × (ECMO pump speed/2350)^2.04^, where female = 0 and male = 1 and V (L) = 41.

Table 2 summarizes the model-derived final population PK parameters for remifentanil with its associated interindividual variability and median parameter estimates with 95% CIs from 5000 bootstrap replications. The PK estimates obtained from the final model were similar to those generated from bootstrap replications, indicating good precision in the final model.

**Table 2:** Final pharmacokinetic model parameters of remifentanil in critically ill patients receiving venoarterial extracorporeal membrane oxygenation.

Parameters	Final model	Bootstrap (n = 5000)^a^	
	Population estimate (RSE %) [shrinkage]	Median	95% CI (2.5–97.5%)
**Fixed effects**			
θ_CL_	366 (24)	384	253‒705
θ_V_	41 (21)	39.7	27.8‒83.6
θ_sex_ on CL	0.502 (26)	0.474	0.226‒0.724
θ_ECMO pump speed_ on CL	2.04 (62)	2.14	0.252‒5.09
**Random effects**			
Interindividual variability (ω)^b^			
ω_CL_	0.124 (67) [10]	0.121	0.012‒0.265
Residual variability (σ)			
σ_proportional_	0.387 (18)	0.372	0.186‒0.497
σ_additive_	0.111 ng/ml (31)	0.112 ng/ml	0.015‒0.218

^a^95% CI estimated from 5000 resampled data sets using the final population pharmacokinetic model. ^b^Interindividual variability on volume of distribution (V) was not estimated. TVCL, typical value of clearance (L/h); TVV, typical value of volume of distribution (L); ω_CL_, interindividual variability of clearance; σ_proportional_, proportional residual error; σ_additive_, additive residual error.

**Figure 2:**
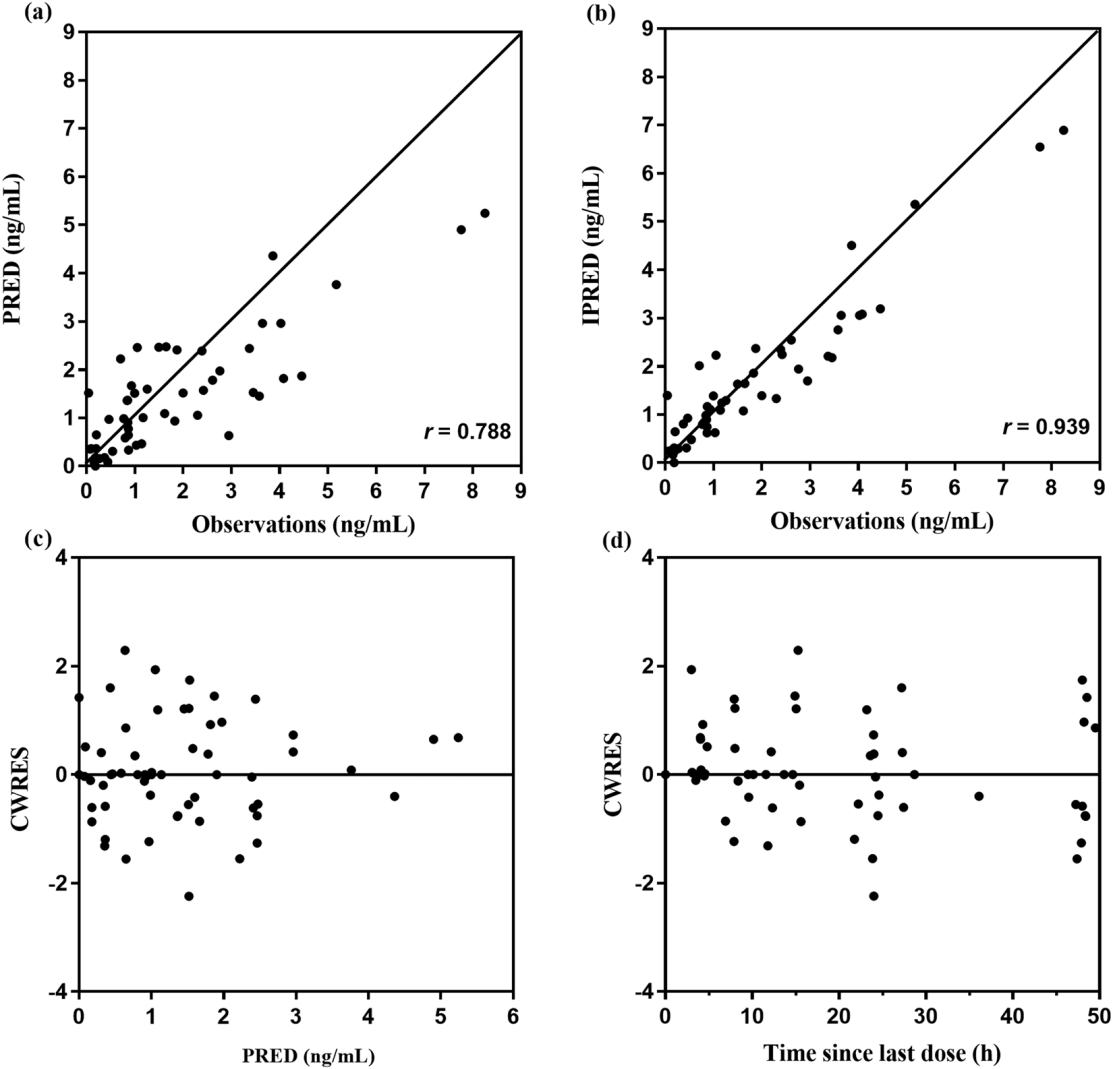
Goodness-of-fit plots of the final population pharmacokinetics (PK) model for continuous remifentanil infusion in critically ill patients on ECMO. Observed remifentanil concentrations vs. (**a**) population-predicted concentrations (PRED) and (**b**) individual-predicted concentrations (IPRED); conditional weighted residuals (CWRES) vs. (**c**) population-predicted concentrations (PRED), and (**d**) time since last dose.

**Figure 3:**
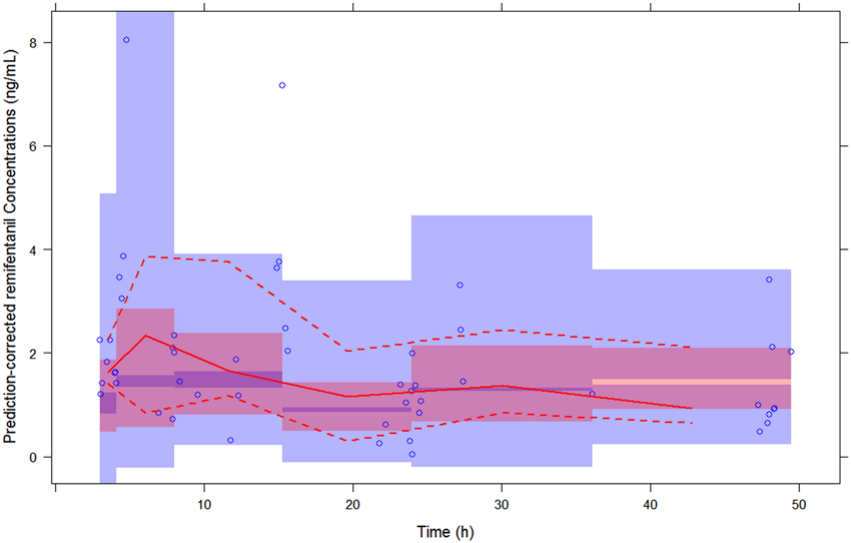
Prediction-corrected visual predictive check of the final population PK model for remifentanil. A thousand simulations were performed. Prediction-corrected observed concentrations are shown as open circles. The middle solid, lower dashed, and upper dashed lines represent the median, 2.5^th^, and 97.5^th^ percentiles for the observed data, respectively. The shaded areas represent a 95% CI for a simulated predicted median, 2.5^th^, and 97.5^th^ percentiles constructed from 1000 simulated datasets of individuals from the original dataset.

Figure 2 shows basic goodness-of-fit plots for the final population PK models for remifentanil. An examination of the goodness-of-plots demonstrated that population prediction and individual population prediction were evenly distributed across the line of identity. The individual population prediction (r = 0.939) showed an improvement over population prediction (r = 0.788), indicating a good model fit. In addition, the conditional weighted residuals were symmetrically distributed around the line of zero (within ± 2 standard deviations of the mean) without an obvious trend, indicating no evidence of model misspecification. Overall, the goodness-of-fit plots show no clear systematic bias in the structural and residual error models. PcVPCs with 95% prediction intervals using the final population PK model are shown in Fig. 3. PcVPC plots showed that most of the observed concentrations were overlaid within 95% of the predictive interval of simulated data, suggestive of the adequate predictive performance of the final model.

### Predicted concentration profiles

**Figure 4:**
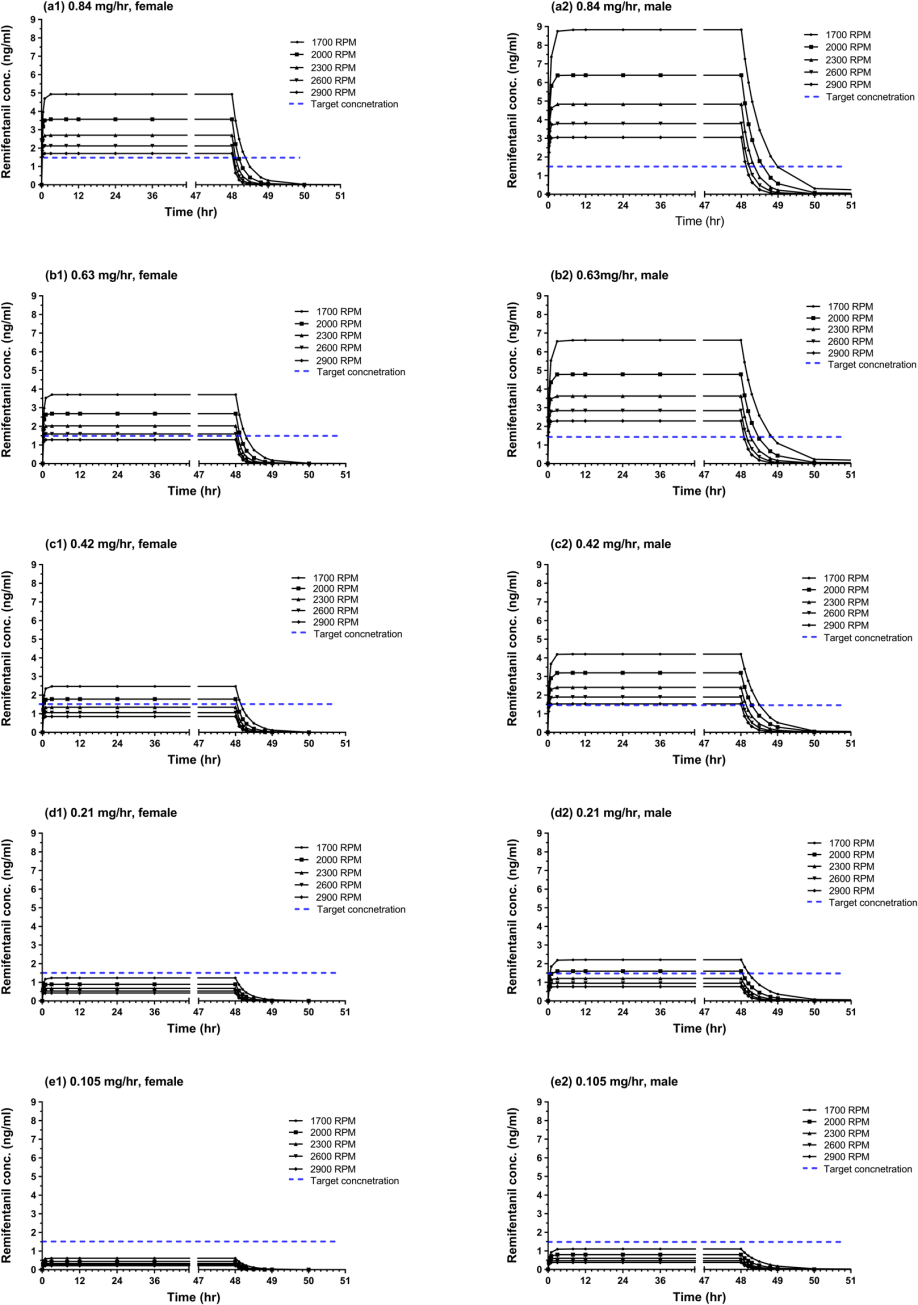
Simulated mean remifentanil concentrations in female vs. male patients with ECMO pump speeds of 1700, 2000, 2300, 2600, and 2900 RPM. (**a1**) 0.84 mg/h in female patients, (**a2**) 0.84 mg/h in male patients, (**b1**) 0.63 mg/h in female patients, (**b2**) 0.63 mg/h in male patients, (**c1**) 0.42 mg/h in female patients, (**c2**) 0.42 mg/h in male patients, (**d1**) 0.21 mg/h in female patients, (**d2**) 0.21 mg/h in male patients, (**e1**) 0.105 mg/h in female patients, and (**e2**) 0.105 mg/h in male patients. Conc, concentration; ECMO, extracorporeal membrane oxygenation; h, hour; RPM, revolution per minute.

Figure 4 shows the simulated remifentanil concentrations affected by sex and ECMO pump speeds (range 1700–2900 RPM) for each dose. According to sex and different speeds of the ECMO pump, patients may receive corresponding doses to ensure 95% maintain remifentanil concentrations ≥1.5 ng/mL^15^ throughout the treatment of VA-ECMO.

1. For female patients: pump speed 1700–2000 RPM, ≥0.42 mg/h; 2000–2900 RPM, ≥0.63 mg/h
2. For male patients: pump speed 1700–2000 RPM, ≥0.21 mg/h; 2000–2900 RPM, ≥0.42 mg/h

## Discussion

In this prospective cohort study, we investigated the population PK of remifentanil in adult patients on VA-ECMO. We found that remifentanil V and CL were increased in adult patients on VA-ECMO compared with previously reported patients not on ECMO, and we identified two significant determinants that affected remifentanil concentrations: sex and ECMO pump speed. The study results implied that patients on VA-ECMO require an increased dose of remifentanil to reach the therapeutic sedation level. Moreover, remifentanil dosing could be adjusted according to sex and ECMO pump speed. To our knowledge, this is the first study to develop a population PK model of remifentanil in adult ICU patients on VA-ECMO. By understanding PK changes that occur during VA-ECMO, clinicians will be able to provide informed decisions regarding appropriate dosing of sedatives to patients on VA-ECMO. This study also added to the paucity of data to guide the optimal dosing of sedatives in adult patients on ECMO with regard to contemporary extracorporeal technology.

In our cohort of VA-ECMO patients, the PK of remifentanil during continuous infusion was well described using a one-compartment model. Remifentanil PK has previously been described with one-compartment^15–17^ or two-compartment models^18,19^. The selection of different models may be because of differences in dosing regimen, sampling scheme, study population, and analytical method. Nevertheless, a model validation showed that PK estimates had good precision and were reasonably unbiased in the present study.

The findings of the present study demonstrated an increased V and CL in ECMO patients compared with previously reported non-ECMO patients. Typical V and CL of continuous remifentanil infusion in our cohort were 41 L and 366 L/h, respectively. The estimates of V and CL were substantially higher than those in healthy volunteers (V range, 18–25 L; CL range, 142–173 L/h) and slightly higher than those in critically ill patients (V range, 19–38 L; CL range, 186–297 L/h)^7,18,20^. This trend of PK alterations was anticipated as V and CL of lipophilic drugs, such as remifentanil, are often reported to be increased in ECMO patients^21^.

An increase in V and CL has been demonstrated in other sedatives administered during ECMO^22^. Ahsman *et al*. have reported that 20 neonates on VA-ECMO showed increased V and CL of midazolam^23^. Potential explanations for increased V include critical illness (e.g., systemic inflammation, sepsis) and drug sequestration in ECMO circuits^10^. The sequestration of drugs occurs when the blood is exposed to a large surface area of circuit membranes during their transit through the circuit, particularly for lipophilic and protein-bound drugs^24^. *Ex vivo* studies on the ECMO system revealed that the extent of drug adsorption increases with lipophilicity and the degree of bound protein^25,26^. The adsorption of remifentanil to the ECMO circuit is anticipated as remifentanil is moderately lipophilic (log *P* = 1.25–1.75) and protein bound (70%)^27,28^. The adsorption of remifentanil may result in drug loss over time (reduced drug concentrations) and, subsequently, therapeutic failure. Other explanations for an increased V include an expanded exogenous blood volume (plasma, albumin or saline) required to prime the ECMO circuit, an altered serum protein concentration, and massive transfusion^4,11,29^. Furthermore, the typical CL of remifentanil in our cohort moderately increased compared with previously reported non-ECMO patients. For drugs previously studied with ECMO, such as midazolam and sildenafil, the reported increase in CL was up to 160%^23,30^. It is likely that CL is increased because the patients had low plasma protein. Decreased plasma protein results in a high fraction of the free drug for rapid clearance from the body. Overall, the introduction of the ECMO system may have influenced the remifentanil PK and concentration levels^25^. Therefore, clinicians should be aware that an increased dose of remifentanil is required to achieve the desired level of sedation. In addition, clinicians should anticipate the need for dose reduction following ECMO removal, owing to a rapid decrease in V and CL.

In the present study, the major determinants influencing on the PK of remifentanil were sex and ECMO pump speed. Remifentanil CL was higher in female patients than in male patients, resulting in reduced remifentanil concentrations in female patients. Females and males differ in body compositions. Females have a greater proportion of body fat and a lower content of body water than males. The differences in body fat compositions may affect the PK of many drugs. For lipophilic drugs such as remifentanil, females appear to have reduced drug concentrations as a result of higher V and possibly higher CL^31^. A previous study conducted with a Chinese population found that female patients showed higher remifentanil CL than male patients, which is consistent with the present study^32^. Yet, there is no conclusive information indicating that true sex differences exist in the PK of remifentanil due to small sample size and uneven sex ratio in the present study. Therefore, a sex-based dosing modification should be further investigated.

In addition, we evaluated the effect of ECMO circuit factors, ECMO flow rates and ECMO pump, on the PK of remifentanil. ECMO pump speed was positively correlated with remifentanil CL, whereas ECMO flow rates were not significantly associated with PK parameters. It is unclear why ECMO pump speed, but not ECMO flow rates, affects the PK of remifentanil. However, the components of the ECMO circuit, particularly the centrifugal pump, can induce drug degradation^33^. At a high pump speed, remifentanil may undergo a high degree of spontaneous degradation, which can result in increased CL and reduced remifentanil concentrations. Although it is challenging to arrive at a definite conclusion based on these data, this finding provides clinicians insight into whether dosing modification of remifentanil-based on the pump speed may be necessary during VA-ECMO.

The simulations from the final population PK model present the predicted remifentanil exposures affected by sex and the ECMO pump speed in VA-ECMO patients by exploring the various dosing scenarios. The clinical implications of our findings were examined to determine appropriate dosing regimens at a target concentration of ≥1.5 ng/mL^15^ for optimal sedation. Considering that the median speed of the ECMO pump was 2350 RPM in the present study, female patients with a minimum dose of 0.63 mg/h and male patients with a minimum dose of 0.42 mg/h achieved a target concentration of ≥1.5 ng/mL in most patients.

There are several limitations in the present study. First, although this study was limited by its small sample size (n = 15), Shekar *et al*. estimated that a minimum of 12 patients would be sufficient for a population PK analysis in ECMO patients^34^. Other previous studies included only 9 children and 15 adult ICU patients to develop the population PK model of remifentanil^15,32^. Second, a sparse sampling scheme and few samples failed to describe the saturation of the ECMO tubing and/or changes in levels following changing of the ECMO components. It also may have affected the precision of PK estimates. Nevertheless, our validation evaluation from the final model demonstrated the good estimates of PK parameters to predict remifentanil concentrations. Third, the absence of a control group precludes the comparison of non-ECMO patients in our cohort. Fourth, patients had limited variability or narrow range in their body weight, limiting the generalizability of the data to other patients. Lastly, the variability of ECMO equipment may have a different influence on the PK of remifentanil, limiting the generalizability to other settings.

Ideally, the management of sedation in ICU patients on ECMO should use a personalized approach in delivering the optimal dosing regimen to each patient. In summary, remifentanil V and CL were increased in VA-ECMO patients compared with previously reported non-ECMO patients and significant determinants affecting the PK of remifentanil are sex and ECMO pump speed. Based on the results of this study, we suggest that clinicians should consider an increased remifentanil dosing during VA-ECMO to achieve the desired sedation level. Despite some limitations, this study serves as an initial step toward understanding the remifentanil PK in adult patients on VA-ECMO and providing the optimization of pharmacotherapy within this patient population. Future studies are warranted to validate our findings from a larger population with the non-ECMO control group. Future studies should also consider describing the attainment of sedation goals using pain and agitation scales to make the data more clinically relevant.

## Methods

### Study design and setting

This prospective, cohort study was undertaken in the cardiovascular ICU of Severance Cardiovascular Hospital, a university-affiliated tertiary hospital in Seoul, South Korea, between January 2015 and December 2016 (ClinicalTrials.gov NCT02581280). The study was approved by the institutional review board (IRB) at Severance Hospital (IRB number 4–2014–0919) and conducted in accordance with the principles of the Declaration of Helsinki. Written informed consent was obtained from each participant or their legally authorized representatives (if the participant lacked the capacity to consent) before any study procedure.

### Patients

Patients were eligible for enrollment if they were aged ≥19 years, received remifentanil during VA-ECMO support, and hospitalized in a cardiovascular ICU. Patients were excluded if they were pregnant or lactating mothers, or were known to be allergic to the remifentanil or any of the remifentanil ingredients, were undergoing treatment drugs that may cause potential drug-drug interaction and change the remifentanil concentrations.

### ECMO system

The ECMO system consists of a centrifugal blood pump with a pump controller (Capiox SP^®^, Terumo Inc., Tokyo, Japan), a polymethylpentene (PMP) circuit (Capiox EBS® with X coating, Terumo Inc., Tokyo, Japan) with poly-(2-methoxyethyl acrylate) (PMEA) polymer-coated polyvinylchloride (PVC) tubing and an air-oxygen mixer (Secrist Ind., Anaheim, CA, USA). The centrifugal blood pump was set at an initial blow flow of 3.0–4.0 L/min. ECMO equipment was implanted using percutaneous femoral peripheral cannulation with a 17-Fr arterial cannula (BioMedicus Medtronic Inc., Minneapolis, MN, USA) and a 21-Fr venous cannula (BioMedicus multistage femoral venous cannula).

### Study procedures

In this observational study, remifentanil and all other concomitant medications were administered at the discretion of the treating physician and were not affected by study procedures. Remifentanil (Ultiva^®^, GlaxoSmithKline, Brentford, UK) was administered via continuous infusion and maintained at a fixed infusion rate unless a change in sedation level was detected, as part of the routine course of therapy. No patients required the bolus injection of remifentanil during the study.

The following demographic and clinical data from the electronic medical records were collected: age, sex, body weight, BMI, remifentanil dose and infusion rate, duration of ECMO, ECMO flow rate, ECMO pump speed, use of continuous renal replacement therapy (CRRT), serum creatinine (SCr), total protein, and temperature.

PK sampling was performed during ECMO support. Sparse PK samples for measurement of remifentanil concentrations (3 mL) were drawn from dwelling arterial lines at 8–12 (T1), 24 (T2), and 36–48 (T3) h. If remifentanil was discontinued during VA-ECMO, serial PK samples were collected immediately before the discontinuation and at 5, 10, 15, 25, 30, and 40 min. Blood samples were collected into EDTA-coated tubes, which were directly placed on ice, followed by immediate centrifugation and addition of formic acid to EDTA plasma. The supernatants were stored at −80 °C until subsequent assay. The remifentanil assay procedures were followed as previously described^35^.

### Remifentanil concentration assay

Remifentanil concentrations were analyzed using a validated liquid chromatography-tandem mass spectrometry instrument (Shimadzu Inc. Corp, Kyoto, Japan). Plasma samples were denatured with acetonitrile consisting of 1 μg/mL chlorpropamide (IS, internal standard). The mixture was vortexed and centrifuged. A chromatographic gradient was applied using a mixture of acetonitrile and water (20:80, v/v)with 0.1% formic acid. The assay was validated between 0.05 and 500 ng/mL with an inter-assay coefficient of variation <15% except for the lower limit of quantification (LLOQ). The inter- and intra-assay coefficients of variation were 5.6% and 0.6%, respectively. The LLOQ for remifentanil was 0.05 ng/mL. The calibration curve was linear between 0.05 and 500 ng/mL with an *R* ^2^ of 0.988.

### Population PK modelling

The population PK model for the concentration-time data for remifentanil concentrations was developed by using NONMEM^®^ version 7.3. (ICON Development, Ellicott City, MD, USA) bundled with a gfortran compiler and Perl-Speaks to NONMEM (PsN) toolkit^36^. The first-order conditional estimation with the interaction (FOCE INTER) approach was used to estimate typical population PK parameters, interindividual variability, and residual variability throughout the model development process. Data visualization and output evaluation were performed using Pirana^®^ version 2.9.2^37^ and Xpose^®^ version 4.0 (http://xpose.sourceforge.net) in R^®^ version 3.2.1 (http://www.r-project.org)^38^.

### Structural and model development

One- and two-compartment models were evaluated as potential structural PK models according to previous studies^15,16,18,19,39^. For interindividual variability (η) of population PK parameters, an exponential variance model was evaluated and assumed to follow a log-normal distribution with a mean of zero and a variance of ω^2^. For residual variability (ε), additive, proportional, and combined additive and proportional error models were evaluated and assumed to be normally distributed with a mean of zero and a variance of *σ* ^2^.

Model development and selection were based on the minimum objective function value (OFV), minimization successful, the plausibility of model-derived PK parameter estimates relative standard errors (RSE), and a visual inspection of graphical goodness-of-fit. A reduction in the OFV of > 3.84 (*x* ^2^ distribution, the degree of freedom = 1) was considered statistically significant (*p* < 0.05). A goodness-of-fit was assessed by the correlation coefficient, and visual inspection of the observed concentrations vs. individual predictions (IPRED), observed concentrations vs. population predictions (PRED), and conditional weighted residuals vs. population predictions and time.

### Covariate model

Potential covariates were tested for their influence on the PK parameters of remifentanil. The continuous covariates tested included age (years), body weight (kg), ECMO flow rate (liters per minute, LPM), ECMO pump speed (revolutions per minute, RPM), serum creatinine (mg/dL), and temperature (°C). These covariates were centered using their median values and evaluated using a power, exponential, and linear model. The categorical covariates tested included sex (where female = 0, male = 1) and the presence of CRRT support (where absent = 0, presence = 1), which were evaluated using a power, exponential, and proportional model. Covariates that were biologically or clinically plausible, reduced the OFV by > 3.84 (*p* < 0.05, *x* ^2^ distribution, the degree of freedom = 1), decreased residual variability, and improved the goodness-of-fit plots were included in the final model.

### Model diagnostics and validation

The nonparametric bootstrap method (n = 5000) was performed to evaluate the precision and stability of the final model. The median with 95% confidence intervals (CI) (2.5–97.5 percentiles) for the bootstrap replicates was generated and compared with the PK parameter estimates from the final model. Moreover, prediction-corrected visual predictive checks (pcVPCs) were performed for model validation^40^. A thousand simulated datasets of individuals from the original dataset were graphed and compared with prediction-corrected observed concentrations of a continuous infusion of remifentanil, which were overlaid with the 95% CIs of the simulated 5^th^, 50^th^, and 95^th^ percentile curves.

### Simulations

Monte Carlo simulations (n = 5000) were performed using the PK parameters of the final model to examine the effect of sex and the ECMO pump speed on predicted remifentanil concentrations and propose the dosing regimen. The ECMO pump speeds simulated were 1700, 2000, 2300, 2600, and 2900 RPM in female vs. male patients with different dosing regimens. We assumed that remifentanil was continuously infused for 2 days with the following infusion rates: 0.84, 0.63, 0.42, 0.21, and 0.105 mg/h. While interpreting the simulation results, a target concentration ≥1.5 ng/mL was considered for optimal sedation according to preliminary evidence^15^.

### Data availability

The datasets generated during and/or analyzed during the current study are available from the corresponding author on reasonable request.
